# High Power CW Wattmeter Calibration at NIST

**DOI:** 10.6028/jres.097.031

**Published:** 1992

**Authors:** Gregorio Rebuldela, Jeffrey A. Jargon

**Affiliations:** National Institute of Standards and Technology, Boulder, CO 80303

**Keywords:** automated, calibration, cascaded, continuous wave, coupler, high power, measurement, transfer, uncertainty, wattmeter

## Abstract

The National Institute of Standards and Technology has established a measurement capability to support high power systems and devices. The automated wattmeter calibration system operates at power levels of 1 to 1000 W for frequencies from 1 to 30 MHz and 1 to 500 W from 30 to 400 MHz. A cascaded coupler technique is used to extend power measurements to high levels which are traccable to a 10 mW standard thermistor mount. This technique uses an arrangement of nominal 10, 20, 30, 40, and 50 dB couplers with sidearm power meters. The initial step transfers the calibration of the 10 mW standard to the 10 dB coupler/power meter. The standard is then replaced with a wattmeter to be calibrated. RF power is increased 10 dB and the calibration is transferred to the adjacent 20 dB eoupler/power meter. This sequence is repeated with the remaining coupler/power meters until the wattmeter is calibrated at the desired power levels and frequencies. Power ratios calculated from simultaneous power measurements made at each transfer are used to calculate the incident power at the wattmeter. Due to nonideal components, corrections are made for nonlinearities, mismatch, and other errors. Two types of wattmeters have been evaluated at selected frequencies and power levels. Total uncertainties are based on the random and systematic components.

## 1. Introduction

There has been a recent interest in and demand for improved high power calibrations to support new and more accurate high power systems and devices being developed by industry. NIST has established a measurement capability to provide a traceability for continuous wave (cw) high power measurements. This paper describes the system, measurement scheme, calibration results and uncertainty analysis of the calibrations performed on different types of high power wattmeters.

## 2. System Description

**Fig. 1:**
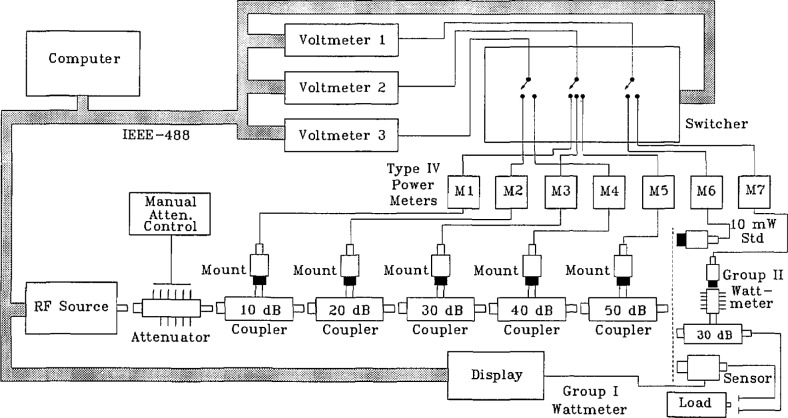
Block diagram of NIST high power cw wattmeter calibration system.

A diagram of the system is shown in Fig. 1. The rf source provides a stable rf signal at power levels of 1 to 1000 W for frequencies from 10 to 30 MHz, and 1 to 500 W from 30 to 400 MHz [1]. The frequency and output power are controlled by software. A closed-loop feedback arrangement maintains the output power within ±0.005 dB. The rf power path is switched to one of three test output ports depending on the frequency.

Since the source delivers a minimum of 1 W and the initial two calibration stages are made at 10 and 100 mW, an in-line attenuator is inserted between the source and the 10 dB coupler to reduce the power to the required levels. This latching attenuator has a range of 1 to 31 dB in 1 db steps and is controlled manually.

The cascaded coupler arrangement is composed of nominal 10, 20, 30, 40, and 50 dB directional couplers with sidearm power meters connected to digital voltmeters. Five coupler/power meters are required to transfer powers from 10 mW to 1000 W in 10 dB steps. Each sidearm power meter, composed of a thermistor mount in conjuction with a NIST Type IV bridge, is connected to a digital voltmeter to measure rf powers within the bandwidth of the thermistor mount. The switcher connects each voltmeter to one of seven power meters depending on the stage of the calibration. A calibrated thermistor mount serves as the 10 mW reference for extending measurements to higher levels.

Measurements are performed on two types of wattmeters. Group I includes three similar commercial units that measure rf power directly using diode power sensors. These sensors, used in conjunction with a power meter as a display, are microprocessor-based, each carrying its own wideband calibration constants in a self-contained nonvolatile memory. Since the calibration data are stored in the sensor, any sensor may be used with any power meter. Group II consists of two 30 dB couplers each with a manually switched, 0–31 dB step attenuator and thermistor mount on the side-arm. The attenuation is determined by the rf power incident on the coupler.

The computer controls the rf source, the digital voltmeters, and the switcher, and handles the data acquisition and processing through an IEEE-488 bus.

## 3. Measurement Methods

### 3.1 Cascaded Coupler Technique

At NIST, the measurement of rf power below 1 GHz has been limited to 10 mW with thermistor mounts at uncertainties of ±0.5% or better. A cascaded coupler technique, developed by K. E. Bramali [2], extends measurements to higher levels which are traceable to a 10 mW standard. Each stage is summarized below.

#### Stage 1

**Fig. 2:**
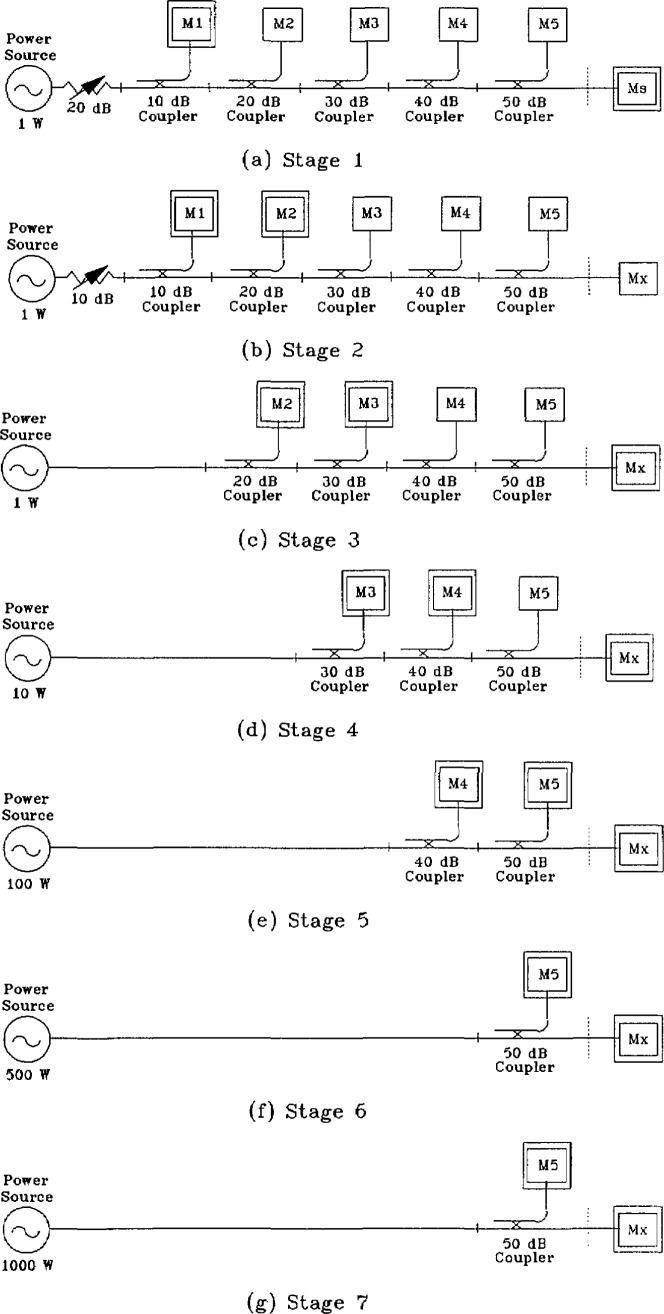
Cascaded coupler arrangements for power transfer from 10 mW to 1000 W.

The 10 mW standard is connected to the cascaded coupler arrangement as shown in Fig. 2(a). Since the source delivers a minimum of 1 W, an in-line attenuator is inserted between the source and the input of the 10 dB coupler/thermistor mount combination to prevent any damage to the reference standard. With the attenuator set to 20 dB, approximately 10 mW are applied to the reference standard. Simultaneous readings are taken on M_1_ and M_S_. The power on M_1_ is nominally 1 mW and due to the insertion loss of the coupler chain, M_S_ will indicate slightly less than 10 mW.

#### Stage 2

The 10 mW standard is replaced with the wattmeter, M_X_, to be calibrated as shown in Fig. 2(b). If the wattmeter is from Group II, its attenuator is set to 0 dB, and the rf power is increased 10 dB to 100 mW, by setting the in-line attenuator to 10 dB. Simultaneous readings result in a nominal 10 mW on M_1_ and 1 mW on M_2_. The main-arm output power, *P*_L_, incident on the wattmeter is approximately 100 mW and is given by

$$P_{L}=\frac{{P_{1}}^{\prime }}{P_{1}}\frac{P_{S}}{K_{B}},$$ (1)

where *P*_1_ is the reading of the sidearm power meter of the 10 dB coupler/thermistor mount, M_1_, when the calibration was transferred from the 10 mW standard, ${P_{1}}^{\prime }$ is the reading of M_1_ when it was used to transfer the calibration to M_2_, and *P*_S_ is the reading of M_S_ from the first stage. The calibration factor, *K*_B_, of the 10 mW standard is defined as the ratio of the substituted dc power in the thermistor mount to the cw rf power incident upon it.

Equation (1) is true only if the impedances of the power standard and the wattmeter are equal. Since they are not, the expression is modified to include the effects of mismatch [3].

$$P_{L}=\frac{{P_{1}}^{\prime }}{P_{1}}\frac{P_{S}}{K_{B}}\frac{{\left|1-\Gamma_{\text{GE}}\Gamma_{S}\right|}^{2}}{{\left|1-\Gamma_{\text{GE}}\Gamma_{X}\right|}^{2}},$$ (2)

where *Γ*_S_ and *Γ*_X_ are the reflection coefficients of the standard and wattmeter, respectively. The factor, *Γ*_GE_, is defined by Engen [4] as the equivalent generator reflection coefficient, and is given in terms of the coupler chain’s scattering parameters,

$$\Gamma_{\text{GE}}=S_{22}-\frac{S_{21}S_{32}}{S_{31}},$$ (3)

where the input of the 10 dB coupler is port 1, the output of the 50 dB coupler is port 2, and the side-arm of the 10 dB coupler is port 3.

#### Stage 3

The in-line attenuator and the 10 dB coupler/thermistor is removed, as shown in Fig. 2(c). The calibration of the 20 dB coupler/thermistor is not affected since a directional coupler has the property that the power split between the main and sidearm is independent of the source characteristics [5].

The source is set to 1 W, a 10 dB increase from the previous stage, and simultaneous readings are taken on M_2_, M_3_, and M_X_. The reading on M_2_, referred to as ${P_{2}}^{\prime }$, is about 10 mW, while *P*_3_, the reading on M_3_, is approximately 1 mW. The main-arm output power, *P*_L_, is about 1 W and is given by

$$P_{L}=\frac{{P_{1}}^{\prime }}{P_{1}}\frac{{P_{2}}^{\prime }}{P_{2}}\frac{P_{S}}{K_{B}}MM,$$ (4)

where

$$MM=\frac{{\left|1-\Gamma_{\text{GE}}\Gamma_{S}\right|}^{2}}{{\left|1-\Gamma_{\text{GE}}\Gamma_{X}\right|}^{2}}.$$ (5)

At this level, the calibration factor, *K*_f1_, of the Group I wattmeter is defined as

$$K_{f1}=\frac{P_{X}}{P_{L}},$$ (6)

where *P*_X_ is the reading on the wattmeter’s display. The calibration factor, *K*_f2_, of the Group II wattmeter is defined as

$$K_{f2}=\frac{P_{L}}{P_{X}},$$ (7)

where *P*_X_ is the substituted dc power of the wattmeter’s thermistor mount. In both cases, *P*_L_ is the rf power incident on the wattmeters and is calculated at each rf power level.

#### Stage 4

The 20 dB coupler/thermistor is removed as shown in Fig. 2(d), and the source power is increased 10 dB, to about 10 W. Simultaneous readings are taken on M_3_, M_4_, and M_X_. The reading on M_3_, called ${P_{3}}^{\prime }$, is about 10 mW and *P*_4_, the reading on M_4_, is about 1 mW. The wattmeter is calibrated at this level using either Eq. (6) or (7), depending on the wattmeter. The main-arm output power, *P*_L_, is nominally 10 W and is given by

$$P_{L}=\frac{{P_{1}}^{\prime }}{P_{1}}\frac{{P_{2}}^{\prime }}{P_{2}}\frac{{P_{3}}^{\prime }}{P_{3}}\frac{P_{S}}{K_{B}}MM.$$ (8)

#### Stage 5

The 30 dB coupler/thermistor is removed as shown in Fig. 2(e). If the wattmeter is from Group II, its attenuator is set to 10 dB to prevent damage to its thermistor mount from subsequent increases of power. The source power is increased 10 dB, to about 100 W and simultaneous readings are taken on M_4_, *M*_5_, and M_X_. The reading on M_4_, called ${P_{4}}^{\prime }$, is about 10 mW, and *P*_5_ is about 1 mW. The main-arm output power, *P*_L_, is nominally 100 W and is given by

$$P_{L}=\frac{{P_{1}}^{\prime }}{P_{1}}\frac{{P_{2}}^{\prime }}{P_{2}}\frac{{P_{3}}^{\prime }}{P_{3}}\frac{{P_{4}}^{\prime }}{P_{4}}\frac{P_{S}}{K_{B}}MM.$$ (9)

#### Stage 6

The 40 dB coupler/thermistor is removed as shown in Fig. 2(f). If it is desired to calibrate the wattmeter between 100 and 1000 W, such as 500 W, the rf power is increased by 7 dB. If the wattmeter is from Group II, its attenuator is set to 17 dB before applying rf power. Simultaneous readings are taken on M_5_, called ${P_{5}}^{\prime }$, and M_X_. The main-arm output power is given by

$$P_{L}=\frac{{P_{1}}^{\prime }}{P_{1}}\frac{{P_{2}}^{\prime }}{P_{2}}\frac{{P_{3}}^{\prime }}{P_{3}}\frac{{P_{4}}^{\prime }}{P_{4}}\frac{{P_{5}}^{\prime }}{P_{5}}\frac{P_{S}}{K_{B}}MM.$$ (10)

#### Stage 7

The source power is increased by 3 dB, to 1000 W, using the same configuration as the previous stage. See Fig. 2(g). If the wattmeter is from Group II, its attenuator is set to 20 dB, and simultaneous readings are taken on M_5_, called ${P_{5}}^{\prime \prime }$, and M_X_. The main-arm output power is given by

$$P_{L}=\frac{{P_{1}}^{\prime }}{P_{1}}\frac{{P_{2}}^{\prime }}{P_{2}}\frac{{P_{3}}^{\prime }}{P_{3}}\frac{{P_{4}}^{\prime }}{P_{4}}\frac{{P_{5}}^{\prime }}{P_{5}}\frac{P_{S}}{K_{B}}MM.$$ (11)

The wattmeter is now calibrated at 1, 10, 100, 500, and 1000 W at the desired frequency.

### 3.2 Modifications to the Cascaded Coupler Technique

Since the high power source is limited to 500 W at 30–400 MHz, wattmeters from Group I were calibrated at 1, 10, 100, 300 and 500 W in this frequency band. This still requires seven stages in the calibration although stages 6 and 7 are modified for lower powers. Wattmeters from Group II are rated at 200 W, so they were calibrated at 1, 10, 100 and 200 W which required six stages. When the measurements were taken, a 10 dB coupler was not available, so a 14 dB coupler was used instead. The only modification necessary was to set the in-line attenuator to 6 dB rather than 10 dB at the second stage, so enough power would be applied to the thermistors.

### 3.3 Power Measurements

**Fig. 3:**
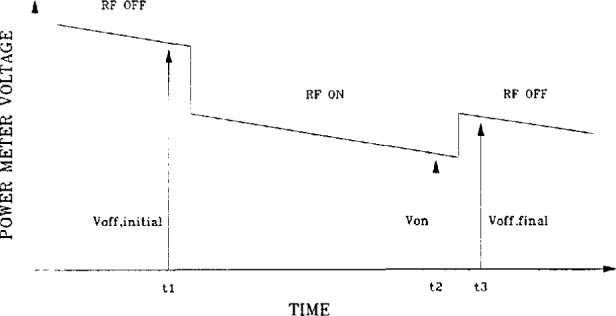
Sequence for measuring power meter dc voltages.

The NIST Type IV power meter does not directly read dc power in watts and must be connected to an external dc voltmeter. The substituted dc power, *P*_dc_, is calculated from measured voltages and is given by

$$P_{\text{dc}}=\frac{{V_{\text{off}}}^{2}-{V_{\text{on}}}^{2}}{R_{0}},$$ (12)

where *V*_off_ is the output voltage with no rf power applied, *V*_on_ is the output voltage with rf power applied, and *R*_0_ is the operating resistance of the thermistor mount. Figure 3 shows the measurement sequence for a power calculation [6]. An initial *V*_off_ is taken; rf power is then applied and *V*_on_ is measured; rf power is removed and a final *V*_off_ is taken. The initial and final dc measurements are used with the *V*_on_ measurement to calculate the power and correct for any mount drift, which is assumed to be linear. The calculated value of *V*_off_ in Eq. (12) is given by

$$V_{\text{off}}=V_{\text{off},i}+\frac{t_{2}-t_{1}}{t_{3}-t_{1}}\left(V_{\text{off},f}-V_{\text{off},i}\right),$$ (13)

where *V*_off,i_ is the voltage reading taken before rf is applied at time *t*_1_, *V*_off,f_ is the voltage taken after rf is removed at time *t*_3_, and *t*_2_ is the time at which *V*_on_ is taken.

## 4. Measurement Results

Measurements were made on both groups of wattmeters at several frequencies and power levels. Group I wattmeters were calibrated at 1, 10, 100, 500 and 1000 W at frequencies from 2 to 30 MHz and at 1, 10, 100, 300 and 500 W at frequencies from 30 to 400 MHz. Group II wattmeters were calibrated at 1, 10, 100 and 200 W at the same frequencies.

The calibration factors for a Group I wattmeter are near unity at all power levels since it measures power directly with a diode detector. A Group I wattmeter has one sensor, denoted Sensor 1, that measures powers at frequencies between 1.8 and 32 MHz and another, Sensor 2, that measures power at frequencies between 25 and 1000 MHz. Sensor 1 was used at frequencies between 2 and 30 MHz, and Sensor 2 was used at frequencies between 35 and 400 MHz. Table 1 lists calibration factors at selected frequencies for three wattmeters from Group I. The calibration factors differ among wattmeters, and the calibration factor at each frequency increases with power, partly due to nonlinearity in the diode detector.

The calibration factors for a Group II wattmeter range from 1,000 to 20,000 due to the 30 dB directional coupler and the attenuator’s setting which is dependent on the power level; 0 dB at 1 and 10 W, 10 dB at 100 W, and 13 dB at 200 W. One wattmeter has a frequency range from 2 to 100 MHz and the other has a range from 100 to 400 MHz. Tables 2 and 3 list the measured calibration factors of the two wattmeters.

## 5. Uncertainty Analysis

### 5.1 Systematic Uncertainty

The factors contributing to the total systematic uncertainty are:

- Uncertainty in the dc voltmeter measurements.
- Uncertainty in the Type IV power meters.
- The dual-element substitution errors associated with the coaxial thermistor mounts.
- Uncertainty in the 10 mW standard mount calibration factor.
- Mismatch uncertainty due to the reflection coefficient of the 10 mW standard mount, the reflection coefficient of the wattmeter/high power load combination, and the equivalent generator reflection coefficient.
- Nonlinearities in the cascaded couplers.
- Uncertainty in the high power source.

#### 5.1.1 Voltmeter Uncertainty

The uncertainty in the individual voltmeter readings can be determined by taking the total differential of the power expression, Eq. (12), which gives

$$dP=\frac{2}{R_{0}}\left(V_{\text{off}}\;dV_{\text{off}}-V_{\text{on}}\;dV_{\text{on}}\right).$$ (14)

The total differential of power, Eq. (14), can be determined by taking the differential of *V*_off_, Eq. (13), which gives

$$dV_{\text{off}}=\left(1-T\right)dV_{\text{off},i}+T\;dV_{\text{off},f},$$ (15)

where

$$T=\frac{t_{2}-t_{1}}{t_{3}-t_{1}}.$$ (16)

The uncertainties, d*V*_off,i_, d*V*_off,f_, and d*V*_on_, in the measured values of *V*_off,i_, *V*_off,f_, and *V*_on_, are based on the voltmeter manufacturer’s specifications.

**Fig. 4:**
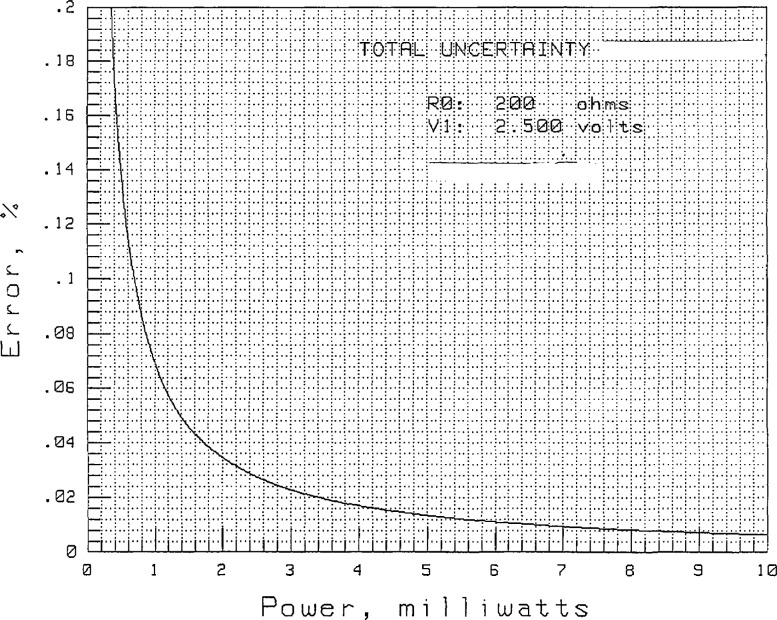
Power measurement uncertainty due to DVM when ratios are taken.

**Fig. 5:**
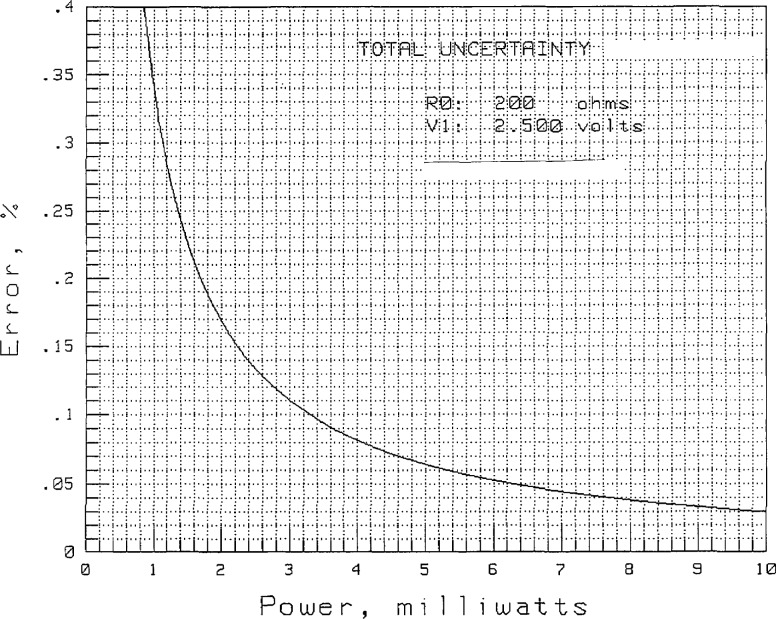
Power measurement uncertainty due to DVM when ratios are not taken.

Figure 4 shows the uncertainty in the power measurement as a function of power level, assuming the coupler sidearm powers, *P*_1_ through *P*_5_ and ${P_{1}}^{\prime }$ through ${P_{5}}^{\prime }$, are ratioed as in the Bramali measurements. Figure 5 shows the uncertainty when a power is not ratioed as in the case of *P*_S_.

The power measurements, ${P_{1}}^{\prime }$ through ${P_{5}}^{\prime }$, are approximately 10 mW, which result in uncertainties of 0.01%. The power measurements, *P*_2_ through *P*_5_, are approximately 1 mW, which result in uncertainties of 0.07%. The measurement of *P*_1_ is about 0.4 mW due to the 14 dB coupler and has an uncertainty of 0.17%.

#### 5.1.2 Type IV Power Meter Uncertainty

The four possible sources of uncertainties internal to the Type IV power meter are the reference resistors, the operational amplifier open-loop gain, input offset voltage, and input bias current. Larsen has shown that the uncertainties due to the Type IV power meters are negligible compared to those of the voltmeters [7].

#### 5.1.3 Dual-Element Uncertainty

The thermistors used in the system are dual-element bolometers. They are nonlinear with power due to the rf-dc substitution error that occurs because the two elements are not identical [8]. The NIST calibration of the effective efficiency is done at 10 mW; therefore, this error is of concern when measurements are made below this power. Direct measurements were performed on similar thermistor mounts [6] resulting in a nonlinearity of about 0.04% at the 1 mW level.

#### 5.1.4 Uncertainty in the Standard Mount Calibration Factor

The uncertainty of the NIST thermistor mount calibration factor, *K*_B_, is approximately ±0.5% in the worst case. The 10 mW standard is recalibrated periodically.

#### 5.1.5 Mismatch Uncertainty

Since the impedances of the standard power meter and the high power load are not equal, mismatch is introduced when the power meter is replaced by the load. The mismatch term, discussed earlier, is given by

$$MM=\frac{{\left|1-\Gamma_{\text{GE}}\Gamma_{S}\right|}^{2}}{{\left|1-\Gamma_{\text{GE}}\Gamma_{X}\right|}^{2}}.$$ (17)

The uncertainty of the mismatch term requires the knowledge of the uncertainties in measuring *Γ*_X_, *Γ*_S_, and the couplers’ scattering coefficients. These uncertainties are given in Table 4. The uncertainty of *Γ*_GE_, which is almost entirely due to the uncertainty of *S*_2_, is ± 0.0034 and is combined with those of the 10 mW standard and wattmeter/load combination to calculate the total mismatch uncertainty. Two different methods were used to analyze the uncertainty.

First, a simulation program was written to calculate the mismatch uncertainty using random values of magnitude and phase, within their respective limits, for the reflection coefficients along with their respective uncertainties. Several hundred trials were performed, resulting in a maximum mismatch uncertainty of ±0.19%.

Second, the mismatch uncertainty was calculated by combining the terms in Eq. (17) in the worst phase with the uncertainties included. The result was a maximum mismatch uncertainty of ±0.2%. The latter method was arbitrarily chosen and its derivation is explained in the Appendix.

#### 5.1.6 Nonlinearity of Couplers

The directional couplers were chosen with power ratings greater than the actual requirements to minimize the power sensitivity of the couplers. Each coupler is rated at least one and one-half times its maximum applied power.

Tests for power nonlinearities were performed on selected couplers at higher powers, and an estimate for the entire coupler chain is approximately ±0.30%.

#### 5.1.7 Uncertainty in the High Power Source

There are several uncertainties due to the radio frequency source, most of which are negligible.

- Harmonics are at least 46 dB below the fundamental signal at the output port, thus having negligible effects.
- Spurious signals are also negligible since they are approximately −60 dBc.
- The frequency uncertainty is approximately ±0.001% due to the internal free-air crystal oscillator of the rf source.
- The rf source amplitude stability is specified by the manufacturer to be ±0.12%.

#### 5.1.8 Overall Systematic Uncertainty

A summary of all the systematic uncertainty components and the total as calculated by the root-sum-square method are shown in Table 5. The overall systematic uncertainty is ±0.67%.

### 5.2 Random Uncertainty

Each of the wattmeters was calibrated five times to determine the repeatability of the measurements. Tests were made at various times of the day over several days to cover as many random factors as possible, including variations of environmental conditions and quality of the connections by the operator. The sample standard deviations were calculated for each meter at all frequencies and power levels. Table 6 lists the standard deviations of the three Group I wattmeters; Table 7 lists the standard deviations of the Group II-A wattmeter (2–30 MHz); and Table 8 lists the standard deviations of the Group II-B wattmeter (30–400 MHz).

**Fig. 6:**
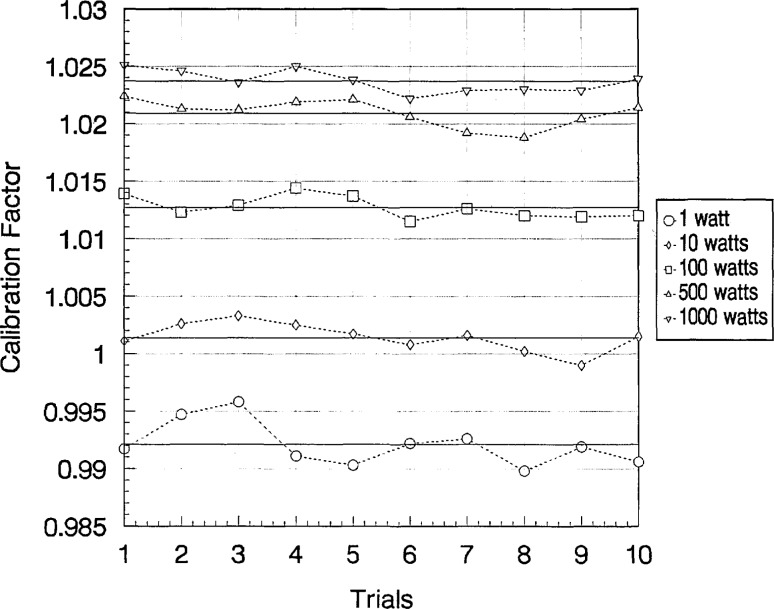
Calculated values of calibration factors (ten trials) for Group I-C wattmeter at 2 MHz and at various power levels. Averages of ten trials shown as solid lines.

**Fig. 7:**
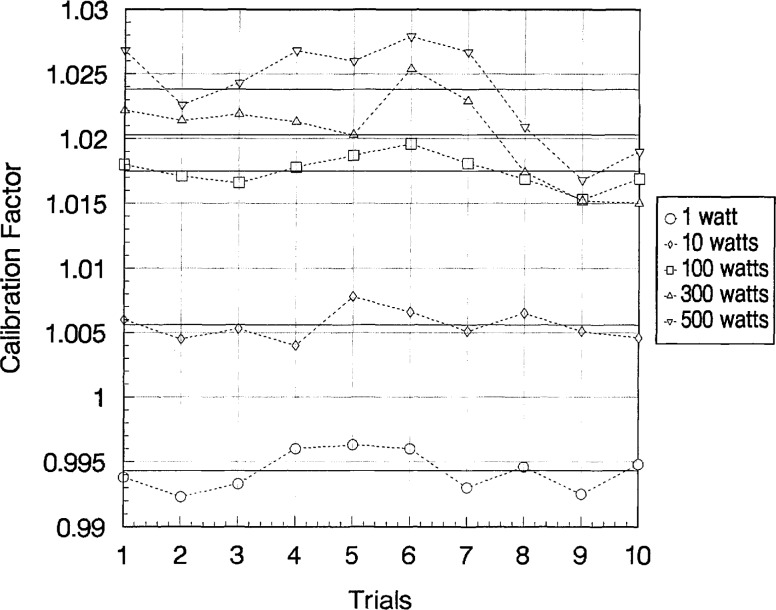
Calculated values of calibration factors (ten trials) for Group I–C wattmeter at 100 MHz and at various power levels. Averages of ten trials shown as solid lines.

**Fig. 8:**
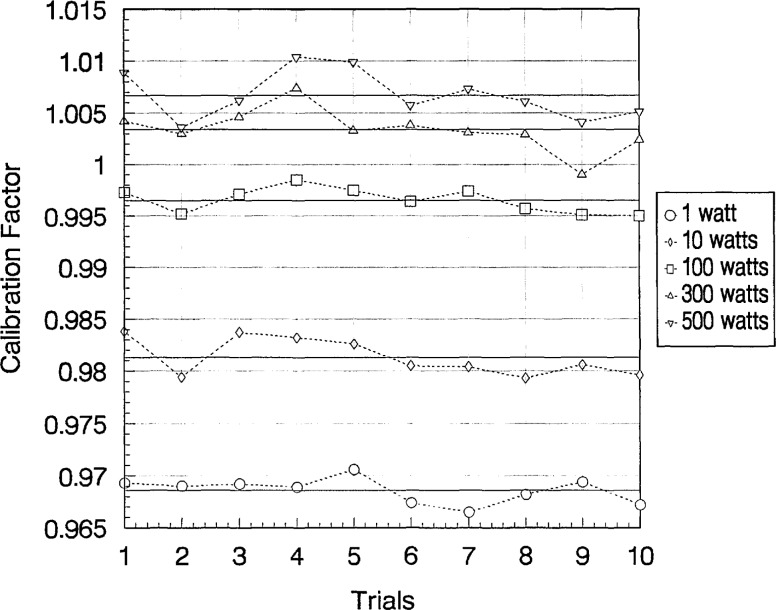
Calculated values of calibration factors (ten trials) for Group I–C wattmeter at 400 MHz and at various power levels. Averages of ten trials shown as solid lines.

Wattmeter C of Group I was calibrated five more times over a 6 month period to determine the long-term stability of the calibration factors. Figures 6, 7, and 8 show the ten measurements at each power level with their averages at 2, 100, and 400 MHz, respectively. Sample standard deviations of the ten trials ranged from 0.07% to 0.66%.

### 5.3 Total Uncertainty

The total uncertainty, *U*_T_, may be calculated by combining the standard deviation, *S*, determined from *N* repeated measurements, with the overall systematic uncertainty, *Δ*, using the equation

$$U_{T}=2\sqrt{\frac{\Delta^{2}}{3}+\frac{S^{2}}{N}}.$$ (18)

Table 9 lists the systematic uncertainty, ranges of values for the random uncertainties, and total uncertainties for each wattmeter.

## 6. Conclusion

The calibration of high power cw wattmeters is accomplished using the cascaded coupler technique. Directional couplers are used to extend the range of low power meters up to the kilowatt range. Although this technique is quite cumbersome and lengthy due to multiple power transfers, the standard deviations are less than 0.66% over a 6 month period for Wattmeter C in Group I. Standard deviations for all other wattmeters vary from 0.03% to 0.80% and are caused largely by the instability of the individual wattmeter. The overall uncertainty limits are 0.77% to 1.05% depending on the type of wattmeter, frequency, and power level. Wattmeters may be used to calibrate a high power source for certifying other wattmeters, thus avoiding the cascaded coupler arrangement and reducing measurement time. However, this introduces another level in the calibration structure, resulting in higher uncertainties.

## Figures and Tables

**Table 1:** Calibration factors of Group I wattmeters

Freq. (MHz)	Power level (W)	Wattmeter A cal. factor	Wattmeter B cal. factor	Wattmeter C cal. factor
2	1	0.9989	0.9929	0.9927
	10	1.0067	1.0062	1.0022
	100	1.0195	1.0196	1.0134
	500	1.0268	1.0285	1.0218
	1000	1.0297	1.0321	1.0244
15	1	1.0014	0.9895	0.9972
	10	1.0087	1.0026	1.0061
	100	1.0198	1.0133	1.0153
	500	1.0240	1.0190	1.0207
	1000	1.0248	1.0202	1.0216
30	1	1.0041	0.9989	0.9950
	10	1.0108	1.0121	1.0022
	100	1.0207	1.0194	1.0118
	500	1.0252	1.0277	1.0167
	1000	1.0269	1.0293	1.0181
40	1	0.9961	0.9955	0.9903
	10	1.0052	1.0047	0.9972
	100	1.0110	1.0142	1.0097
	300	1.0116	1.0161	1.0116
	500	1.0178	1.0220	1.0177
70	1	1.0002	1.0005	0.9857
	10	1.0082	1.0083	0.9965
	100	1.0129	1.0192	1.0094
	300	1.0149	1.0256	1.0153
	500	1.0209	1.0283	1.0185
100	1	1.0059	1.0061	0.9943
	10	1.0126	1.0153	1.0055
	100	1.0180	1.0253	1.0176
	300	1.0235	1.0313	1.0214
	500	1.0271	1.0339	1.0253
125	1	0.9988	0.9960	0.9782
	10	1.0050	1.0068	0.9915
	100	1.0180	1.0238	1.0068
	300	1.0200	1.0300	1.0127
	500	1.0236	1.0318	1.0158
250	1	1.0036	0.9984	0.9758
	10	1.0102	1.0099	0.9905
	100	1.0219	1.0258	1.0045
	300	1.0241	1.0325	1.0112
	500	1.0292	1.0351	1.0125
400	1	0.9939	0.9939	0.9694
	10	1.0025	1.0014	0.9826
	100	1.0135	1.0173	0.9971
	300	1.0169	1.0247	1.0045
	500	1.0230	1.0276	1.0078

**Table 2:** Calibration factors of Group II-A wattmeter

Freq. (MHz)	Power level (W)	Wattmeter A cal. factor
2	1	1105.9
	10	1102.9
	100	11004.2
	200	21747.7
10	1	1139.3
	10	1137.0
	100	11359.1
	200	22506.0
20	1	1167.7
	10	1165.0
	100	11642.0
	200	23119.1
30	1	1189.2
	10	1186.5
	100	11816.8
	200	23586.1
40	1	1224.7
	10	1224.0
	100	12179.5
	200	24047.3
60	1	1272.2
	10	1272.1
	100	12654.4
	200	25036.1
80	1	1320.8
	10	1320.7
	100	13163.1
	200	26174.3
100	1	1378.3
	10	1377.4
	100	13806.0
	200	27561.8

**Table 3:** Calibration factors of Group II-B wattmeter

Freq. (MHz)	Power level (W)	Wattmeter B cal. factor
125	1	1249.6
	10	1247.3
	100	12659.0
	200	25054.4
200	1	1127.0
	10	1122.9
	100	11694.0
	200	23150.8
300	1	1166.4
	10	1162.4
	100	12606.7
	200	24952.1
400	1	1558.4
	10	1553.9
	100	16629.3
	200	33003.9

**Table 4:** Reflection coefficients and uncertainties of mismatch components

Source	Max. value ± uncertainty
Reflection coefficient of 10 mW std.	0.02 ± 0.0030
Reflection coefficient of wattmeter/load combination	0.04 ± 0.0034
Reflection coefficient of equivalent generator	0.12 ± 0.0034
*S*_22_ of coupler chain	0.12 ± 0.0034
*S*_21_ of coupler chain	1.92 ± 0.0050 dB
*S*_32_ of coupler chain	60.95 ± 0.20 dB
*S*_31_ of coupler chain	14.49 ± 0.0095 dB

**Table 5:** Systematic uncertainty components

Uncertainty source	Contribution (%)
dc voltage measurements	
Measurement of *P*_1_	±0.17
Measurement of *P*_2_	±0.07
Measurement of *P*_3_	±0.07
Measurement of *P*_4_	±0.07
Measurement of *P*_5_	±0.07
Measurement of ${P_{1}}^{\prime }$	±0.01
Measurement of ${P_{2}}^{\prime }$	±0.01
Measurement of ${P_{3}}^{\prime }$	±0.01
Measurement of ${P_{4}}^{\prime }$	±0.01
Measurement of ${P_{5}}^{\prime }$	±0.01
Measurement of *P*_S_	±0.03
Dual element of bolometer mounts	
Measurement of *P*_1_	±0.05
Measurement of *P*_2_	±0.04
Measurement of *P*_3_	±0.04
Measurement of *P*_4_	±0.04
Measurement of *P*_5_	±0.04
Power standard calibration factor	±0.50
Mismatch due to reflection coefficients	±0.20
Nonlinearity of cascaded couplers	±0.30
High power source	±0.12

Total (RSS)	±0.67

**Table 6:** Sample Standard deviations of Group I wattmeters

Freq. (MHz)	Power level (W)	Wattmeter A std. dev. *%*	Wattmeter B std. dev. %	Wattmeter C std. dev. %
2	1	0.11	0.13	0.24
	10	0.15	0.12	0.09
	100	0.50	0.72	0.08
	500	0.54	0.75	0.05
	1000	0.59	0.77	0.07
15	1	0.14	0.06	0.18
	10	0.10	0.06	0.10
	100	0.52	0.64	0.09
	500	0.52	0.59	0.07
	1000	0.52	0.63	0.09
30	1	0.08	0.12	0.24
	10	0.12	0.08	0.15
	100	0.51	0.57	0.11
	500	0.52	0.64	0.11
	1000	0.47	0.63	0.08
40	1	0.12	0.19	0.14
	10	0.17	0.57	0.18
	100	0.52	0.77	0.07
	300	0.61	0.70	0.20
	500	0.54	0.75	0.15
70	1	0.16	0.15	0.14
	10	0.20	0.16	0.11
	100	0.54	0.70	0.05
	300	0.51	0.66	0.15
	500	0.56	0.75	0.11
100	1	0.15	0.63	0.18
	10	0.12	0.52	0.15
	100	0.71	0.50	0.08
	300	0.58	0.56	0.07
	500	0.62	0.47	0.18
125	1	0.08	0.11	0.14
	10	0.14	0.08	0.07
	100	0.52	0.29	0.08
	300	0.51	0.32	0.07
	500	0.53	0.35	0.12
250	1	0.06	0.13	0.11
	10	0.12	0.20	0.02
	100	0.42	0.38	0.08
	300	0.46	0.37	0.06
	500	0.45	0.36	0.09
400	1	0.06	0.05	0.07
	10	0.09	0.04	0.19
	100	0.38	0.25	0.12
	300	0.33	0.30	0.17
	500	0.42	0.30	0.28

**Table 7:** Sample standard deviations of Group II-A wattmeter

Freq. (MHz)	Power level (W)	Wattmeter A std. dev. %
2	1	0.20
	10	0.21
	100	0.09
	200	0.07
10	1	0.15
	10	0.10
	100	0.03
	200	0.11
20	1	0.17
	10	0.11
	100	0.13
	200	0.15
30	1	0.20
	10	0.14
	100	0.59
	200	0.46
40	1	0.52
	10	0.54
	100	0.47
	200	0.37
60	1	0.49
	10	0.51
	100	0.20
	200	0.23
80	1	0.40
	10	0.43
	100	0.40
	200	0.25
100	1	0.34
	10	0.40
	100	0.33
	200	0.34

**Table 8:** Sample standard deviations of Group II-B wattmeter

Freq. (MHz)	Power level (W)	Wattmeter B std. dev. %
125	1	0.12
	10	0.10
	100	0.14
	200	0.12
200	1	0.23
	10	0.11
	100	0.18
	200	0.26
300	1	0.43
	10	0.52
	100	0.59
	200	0.80
400	1	0.31
	10	0.13
	100	0.18
	200	0.22

**Table 9:** Systematic uncertainties and ranges of values for the random and total uncertainties of the wattmeters

	Systematic uncertainty (%)	Random uncertainty (%)	Total uncertainty (%)
Group I			
Wattmeter A	0.67	0.06–0.62	0.78–0.95
Wattmeter B	0.67	0.04–0.77	0.77–1.04
Wattmeter C	0.67	0.02–0.28	0.77–0.81
Group II			
Wattmeter A (2–30 MHz)	0.67	0.03–0.59	0.77–0.94
Wattmeter B (30–400 MHz)	0.67	0.10–0.80	0.78–1.05

## 7. Appendix A

Since the impedances of the 10 mW standard and the wattmeter/load are not equal, a mismatch term, *MM*, is introduced [3] and is given by

$$MM=\frac{{\left|1-\Gamma_{\text{GE}}\Gamma_{S}\right|}^{2}}{{\left|1-\Gamma_{\text{GE}}\Gamma_{X}\right|}^{2}},$$ (19)

where *Γ*_X_ and *Γ*_S_ are the reflection coefficients of the wattmeter/load combination and the power standard, respectively, and *Γ*_GE_ is the equivalent generator reflection coefficient.

The reflection coefficients are complex numbers and can be written in the form

$$\Gamma_{\text{GE}}=\left|\Gamma_{\text{GE}}\right|\left(\cos\theta_{\text{GE}}+j\sin\theta_{\text{GE}}\right),$$ (20)

$$\Gamma_{S}=\left|\Gamma_{S}\right|\left(\cos\theta_{S}+j\sin\theta_{S}\right),$$ (21)

$$\Gamma_{X}=\left|\Gamma_{X}\right|\left(\cos\theta_{X}+j\sin\theta_{X}\right)$$ (22)

where θ_GE_, *θ*_S_, and *θ*_X_ are the arguments of the reflection coefficients of the equivalent generator, the power standard, and the wattmeter/load combination.

The mismatch term is simplified and approximated using several steps. First, completing the squares of both the numerator and denominator of Eq. (19) gives

$$MM=\frac{\left|1-2\Gamma_{\text{GE}}\Gamma_{S}+{\left(\Gamma_{\text{GE}}\Gamma_{S}\right)}^{2}\right|}{\left|1-2\Gamma_{\text{GE}}\Gamma_{X}+{\left(\Gamma_{\text{GE}}\Gamma_{X}\right)}^{2}\right|}.$$ (23)

An approximation may be used by deleting the (*Γ*_GE_*Γ*_S_)^2^ and (*Γ*_GE_*Γ*_S_)^2^ terms since their contributions are negligible. This gives

$$MM\approx \frac{\left|1-2\Gamma_{\text{GE}}\Gamma_{S}\right|}{\left|1-2\Gamma_{\text{GE}}\Gamma_{X}\right|}.$$ (24)

Expanding and neglecting the higher order terms, Eq. (24) can be written as

$$MM\approx \frac{1-2\left|\Gamma_{\text{GE}}\right|\left|\Gamma_{S}\right|\cos\left(\theta_{\text{GE}}+\theta_{S}\right)}{1-2\left|\Gamma_{\text{GE}}\right|\left|\Gamma_{X}\right|\cos\left(\theta_{\text{GE}}+\theta_{X}\right)}.$$ (25)

The cosine terms can range in value from −1 to +1. Therefore *MM* has a range

$$MM\approx \frac{1\pm 2\left|\Gamma_{\text{GE}}\right|\left|\Gamma_{S}\right|}{1\pm 2\left|\Gamma_{\text{GE}}\right|\left|\Gamma_{X}\right|}.$$ (26)

With the uncertainties included

$$MM\approx \frac{1\pm 2\left(\left|\Gamma_{\text{GE}}\right|\pm \Delta \left|\Gamma_{\text{GE}}\right|\right)\left(\left|\Gamma_{S}\right|\pm \Delta \left|\Gamma_{S}\right|\right)}{1\pm 2\left(\left|\Gamma_{\text{GE}}\right|\pm \Delta \left|\Gamma_{\text{GE}}\right|\right)\left(\left|\Gamma_{X}\right|\pm \Delta \left|\Gamma_{X}\right|\right)}.$$ (27)

## References

[1] (1990). Installation, Operation and Maintenance Instructions With Illustrated Parts List for Automated Wattmeter Calibration System.

[2] Bramali KE (1971). Accurate Microwave High Power Measurements Using a Cascaded Coupler Method. J Res Natl Bur Stand (US).

[3] Engen GF (1958). Recent Developments in the Field of Microwave Power Measurements at the National Bureau of Standards (US).

[4] Engen GF (1958). Amplitude Stabilization of a Microwave Signal Source.

[5] Beatty RW, Macpherson AC (1953). Mismatch Errors in Microwave Power Measurements. Proc IRE.

[6] Clague FR (1990). Power Measurement System for 1 mW at 1 GHz.

[7] Larsen NT, New A (1976). Self-Balancing DC-Substitution RF Power Meter. IEEE Trans Instrum Meas.

[8] Engen GF, DC-RF A (1964). Substitution Error in Dual-Element Bolometer Mounts. IEEE Trans Instrum Meas.

